# Neuroimmune-driven abrupt neuropsychiatric symptoms in children: preventing pathological fear consolidation

**DOI:** 10.3389/fpsyt.2026.1798369

**Published:** 2026-06-05

**Authors:** Sun Young Yum, Sehee Hwang, Michael Y. Hwang

**Affiliations:** 1 Brain Boutique, Seoul, Republic of Korea; 2 Yonsei University Severance Hospital, Seoul, Republic of Korea; 3 Franklin Delano Roosevelt (FDR) Veterans’ Affairs Medical Center, Montrose, NY, United States

**Keywords:** co-regulation, fear rebalance therapy, fear regulation, fight-flight-or-freeze reactions, neuroinflammation, PANS, scary ride protocol, survival learning

## Abstract

Pediatric Acute-onset Neuropsychiatric Syndrome (PANS) is defined by an abrupt onset of neuropsychiatric symptoms, traditionally viewed through the lens of obsessive-compulsive behaviors. However, it is also characterized by a state of extreme fear. While medical management addresses the biological triggers, this period creates a high-stakes emergency in human fear-learning that remains unaddressed by standard protocols. The current gold standard, exposure and response prevention (ERP), is theoretically mismatched to this unconditioned fear state. How do we help children endure and learn from pre-reflexive fear that is not cognitively mediated, not titratable, involuntary, and remains at peak without habituation? While we wait for the child to become “stable enough” for therapy, primitive learning is already occurring. The question is whether that learning is therapeutically guided or left to raw survival. This conceptual paper proposes Fear Rebalance Therapy (FRT), a theoretical framework requiring empirical validation. It is a developmentally unfolding framework that integrates co-regulation, survival learning and inhibitory learning principles. FRT guides the child to learn the safety of the external world even while the body signals imminent threat. Many of the initial symptoms may be reconceptualized as a threat-dominant physiological state, leading to a cascade of defensive reactions. The PANS index episode represents a critical branch point. Without guided learning, incidental conditioning can lead to chronic, sensitized disability. FRT aims to help children learn to regulate their emotional responses to these internal alarms. This framework proposes that while neuroimmune responses trigger the crisis, the nature of the learning during that crisis may determine trajectory. Early psychological intervention should be an active component of crisis management rather than a post-crisis adjunct to medical care. Importantly, FRT addresses psychological learning processes occurring during the acute episode, not the underlying neuroimmune mechanisms, which remains the domain of medical management.

## 1 Introduction

### 1.1 The neglected psychological emergency in PANS

Pediatric Acute-Onset Neuropsychiatric Syndrome (PANS) presents with a striking paradox. The medical emergency is clear: a neuroimmune process suddenly affecting multiple brain networks, requiring prompt anti-inflammatory treatment. Yet the psychological emergency that accompanies it—catastrophic internal fear—has been consistently described by children and parents while remaining largely unaddressed in the formal literature.

Catastrophic fear is the most prominent and distressing feature of acute PANS. Early clinical descriptions note children who appear “terror-stricken,” “hyperalert,” or in “fight-or-flight states” in the acute phase (1, 2). Families consistently describe overwhelming internal terror as the central experience of crisis onset. Children often summarize their experience simply as: “I’m scared.”

This fear is not focused, symbolic, or testable. It is diffuse and existential. Many describe fears that something terrible will happen to them, their family, or the world, without a specific referent. Adults often translate the experience into familiar diagnostic categories—OCD, anxiety, or separation distress—leaving children feeling misunderstood. As one child explained, clinicians “listen to what they want and then puzzle it together into something” (3). This mismatch can heighten helplessness for both the child and the family, who experience the crisis as frightening and bewildering.

**Figure 1:**
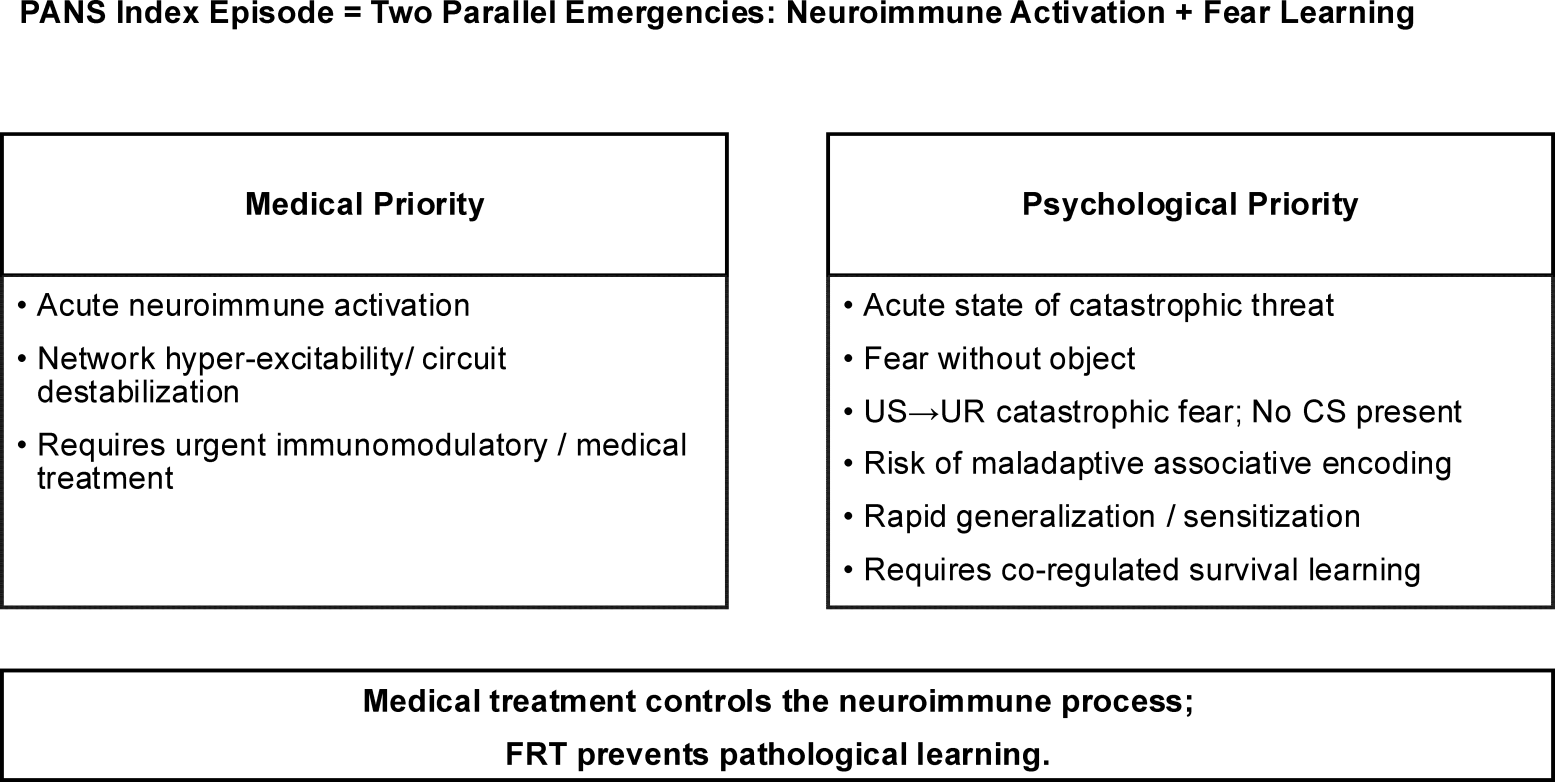
The PANS index episode as dual emergency: medical and psychological priorities. The index PANS episode presents two simultaneous emergencies requiring parallel intervention. The medical priority addresses neuroimmune activation. The psychological priority addresses the emotional experience—acute state catastrophic threat and unconditioned fear—which creates high risk for maladaptive associative learning. Medical treatment controls the underlying neuroimmune process, while Fear Rebalance Therapy (FRT) prevents pathological learning during this vulnerable neurobiological state. Both interventions are necessary: medical treatment alone does not prevent fear sensitization, and psychological intervention alone cannot address the biological driver of the crisis.

Yet fear has not been conceptualized as a primary therapeutic target during the acute phase. It has been overshadowed by the urgency of inflammation control and the familiarity of OCD frameworks (**Figure 1**).

**Figure 2:**
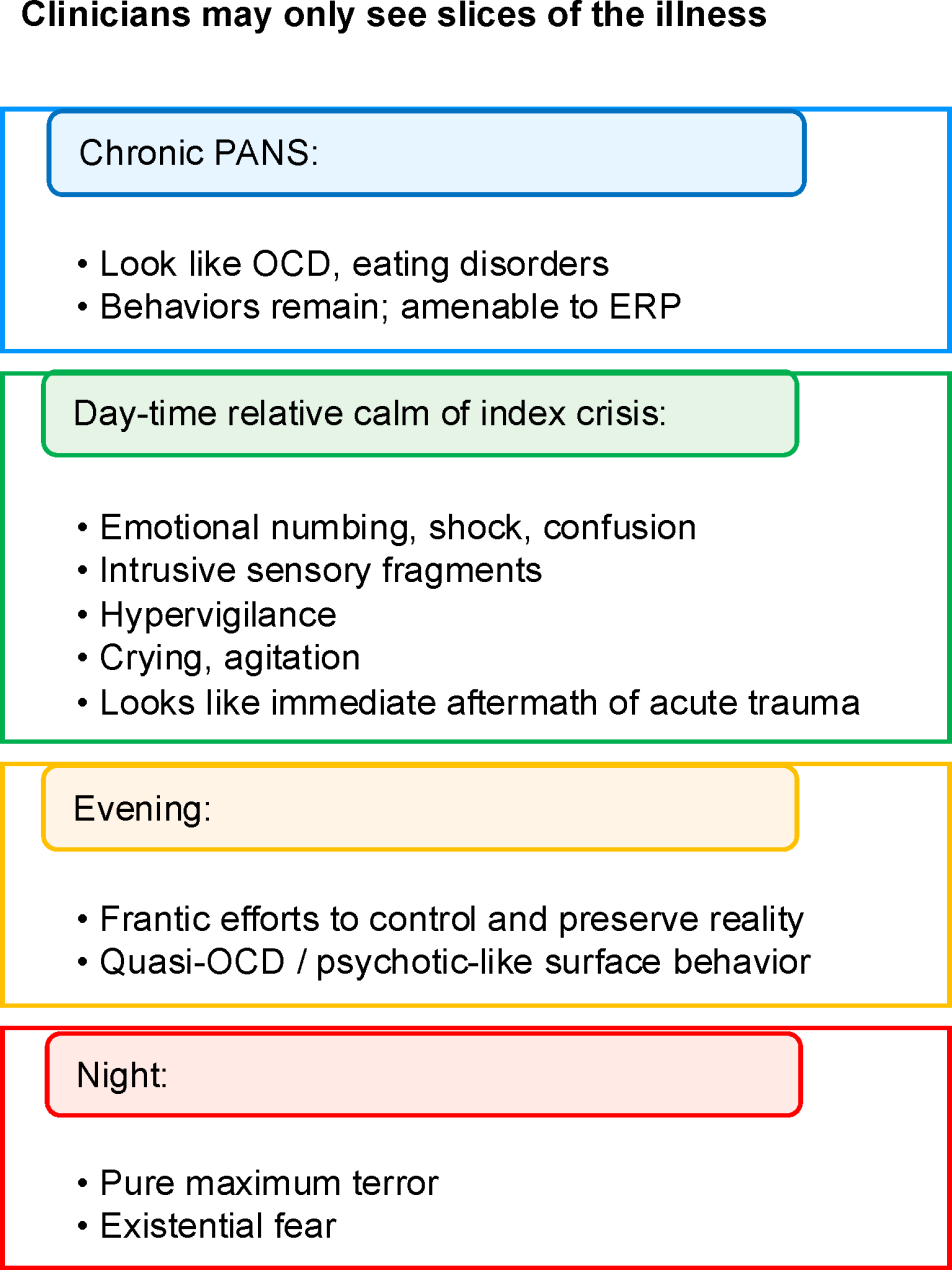
Clinical presentation varies across time: clinicians may only see slices of the illness. PANS presents with dramatically different phenomenology across the circadian cycle and illness trajectory, yet clinicians typically observe only limited time windows. During the index crisis, evening hours may be characterized by frantic efforts to control and preserve reality with quasi-OCD or psychotic-appearing surface behaviors, nighttime brings pure maximum terror and existential fear, and daytime may show relative calm with emotional numbing, shock, confusion, intrusive sensory fragments, hypervigilance, crying, and agitation resembling acute trauma aftermath. Chronic PANS may manifest with behaviors resembling OCD or eating disorders that remain amenable to standard exposure and response prevention (ERP). Clinicians conducting office-hour evaluations may never witness peak nocturnal terror, leading to underestimation of fear severity and potential misdiagnosis. Understanding this temporal heterogeneity is essential for accurate assessment and appropriate intervention planning.

Why this neglect? Most clinicians—even those with extensive PANS experience—have never witnessed the index crisis at peak intensity. Children typically arrive at clinics days to weeks after onset, past the acute phase. The period of maximum fear is brief, occurs at home, and peaks at night. Children appear dramatically different during nocturnal terror, evening attempts to maintain safety, daytime office hours, and aftermath of crisis. Without direct exposure to the nocturnal window, clinicians may underestimate the centrality of fear or view it as secondary to more observable symptoms (**Figure 2**).

### 1.2 The neuroimmune index crisis as unconditioned alarm

The acute fear states of PANS arise from neuroimmune processes that abruptly causes brainwide disruptions (1, 2, 4). The activation of the threat-regulatory circuits produces unconditioned alarm—fear without a conditioned stimulus (CS), but with a real threat source within the brain.

This differs fundamentally from classic OCD, where a CS (contamination cue, checking trigger) generates fear of an imagined catastrophe (unconditioned stimulus; US). The distinction matters because unconditioned fear follows a different learning pathway than conditioned fear. The child’s initial crisis is not a psychiatric disorder that has gradually built associative learning over time; it is biological alarm overwhelming systems responsible for evaluating and regulating emotional experience. The acuity of onset reflects an acute shift in brain state, most likely reflecting changes in the neuroimmune environment.

### 1.3 Current standard of care requires what PANS doesn’t have

Cognitive behavioral therapy (CBT), particularly Exposure and Response Prevention (ERP), is the recommended psychological treatment for PANS. Expert consensus statements advise implementing ERP “as early as possible” (4). But ERP depends on one essential element that PANS onset phase does not have: a CS.

ERP works by systematically exposing a child to their fear trigger: a doorknob, a number, a thought about harm. The therapist constructs a hierarchy, starts with manageable steps, and carefully titrates the dose. The child learns: I touched the doorknob and nothing happened.

In the index PANS crisis, there is no doorknob. The child isn’t afraid of something. They are experiencing raw alarm—neuroimmune activation has hijacked the brain’s threat systems and flooded them with a danger signal that has no external source. There is no CS to expose them to. The feared outcome is not imagined—it is the biological assault itself.

This is not a minor technical problem. It is a categorical incompatibility. ERP requires: a CS (PANS has none), graduated exposure (children are already at maximum exposure), a tolerable arousal window (fear exceeds what the system can process), habituation during exposure (impossible while the threat remains).

**Figure 3:**
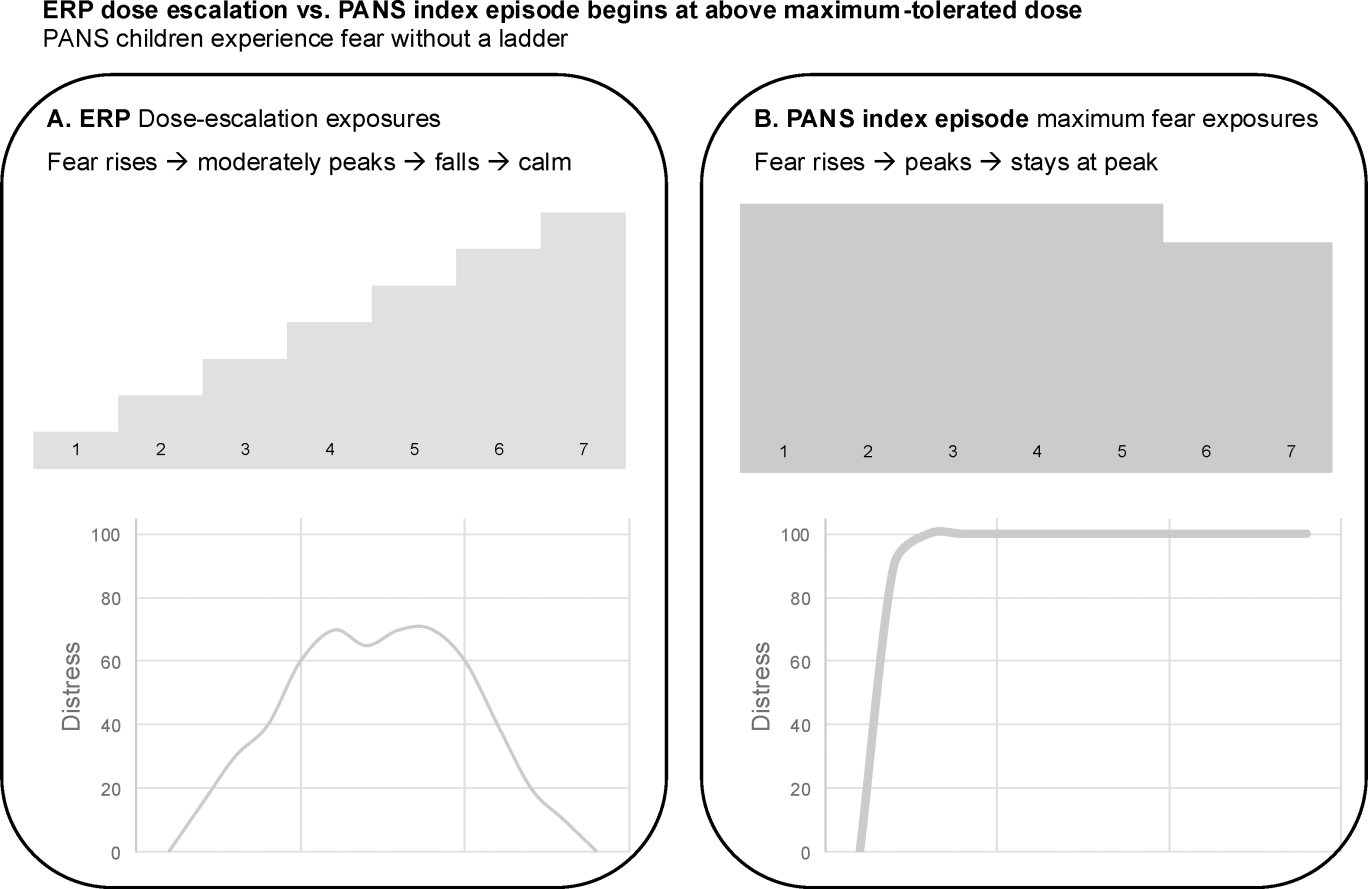
Exposure dose comparison: ERP hierarchies vs. PANS index crisis. Standard exposure and response prevention (ERP) utilizes systematic dose escalation along fear hierarchies. Across sessions, level of distress gradually increases as the patient voluntarily engages with progressively more challenging stimuli. Within each session, fear rises, moderately peaks, and falls as habituation occurs (Panel **(A)**). The PANS index crisis presents an entirely different exposure profile (Panel **(B)**): the child begins at above-maximum-tolerated dose from onset. Fear rises to peak intensity immediately and persists at maximum as long as neuroinflammation remains active. There is no gradual escalation, no dose-response titration, and no within-session habituation. The child experiences involuntary exposure at catastrophic intensity without a fear ladder. Adding exposure tasks to the PANS index episode will likely exceed the child’s tolerable capacity.

Applying ERP “as early as possible” means applying a treatment designed for conditioned fear to unconditioned alarm (**Figure 3**).

### 1.4 Why this matters: the learning environment of crisis

Expert consensus does acknowledge children may be “too acutely ill” for ERP initially (4). A survey of nearly 700 parents similarly found that medical treatment was often needed to precede CBT (5). Published ERP trials enrolled children past the acute stage (6–8). In practice, the index crisis is managed through crisis support, medication trials, and watchful waiting.

But this framing misses something critical: the acute crisis is not a therapy dead zone. It is the most potent learning environment the child will ever experience.

During this window, the child undergoes involuntary exposure to maximum-intensity fear for hours without therapeutic scaffolding. They are actively learning—encoding predictions, forming associations, and interpreting what is happening to them while their fear system screams that survival is at stake.

The question is not whether learning occurs. The question is: what gets learned?

Some children emerge from this crisis and eventually respond well to ERP. Others develop chronic fear sensitization, hypervigilance, and rigid conditioned fear structures that persist long after the neuroinflammation resolves. The difference may lie in what was encoded during those first hours and days—before any therapist entered the picture.

### 1.5 Aim of this paper

This paper examines the index PANS crisis as a dual emergency—acute neuroimmune brain state shift requiring medical intervention and unconditioned fear requiring psychological containment. It proposes Fear Rebalance Therapy (FRT), a developmental framework for supporting fear learning at the onset of PANS. The goal is to intervene in the early psychological environment of the index crisis to prevent maladaptive associative learning, reduce the risk of chronic emotional sensitization, and help children recover without developing conditioned fear structures that later require ERP.

## 2 Theoretical foundation: unconditioned fear and survival-based learning

### 2.1 The learning architecture of acute PANS

The index PANS episode presents a categorically different learning architecture than classic OCD. In classic OCD, fear follows a predictable sequence: CS (contamination cue) → US expectancy (I will get sick) → fear reaction. The rituals and avoidance are conditioned responses built over time through associative learning.

In acute PANS, there is no such sequence. Fear erupts without warning or trigger. The symptoms—rituals, proximity-seeking, sleep refusal, hypervigilance—are not conditioned avoidance behaviors. They are reflexive defensive reactions to a perceived existential threat—what Baldwin describes as the neurobiology of survival, in which existential alarm engages primitive encoding mechanisms that operate outside reflective, declarative learning (9). A child refusing sleep because “the world will end” is not avoiding a learned fear trigger. She is attempting to prevent annihilation as her fear system signals imminent danger.

**Figure 4:**
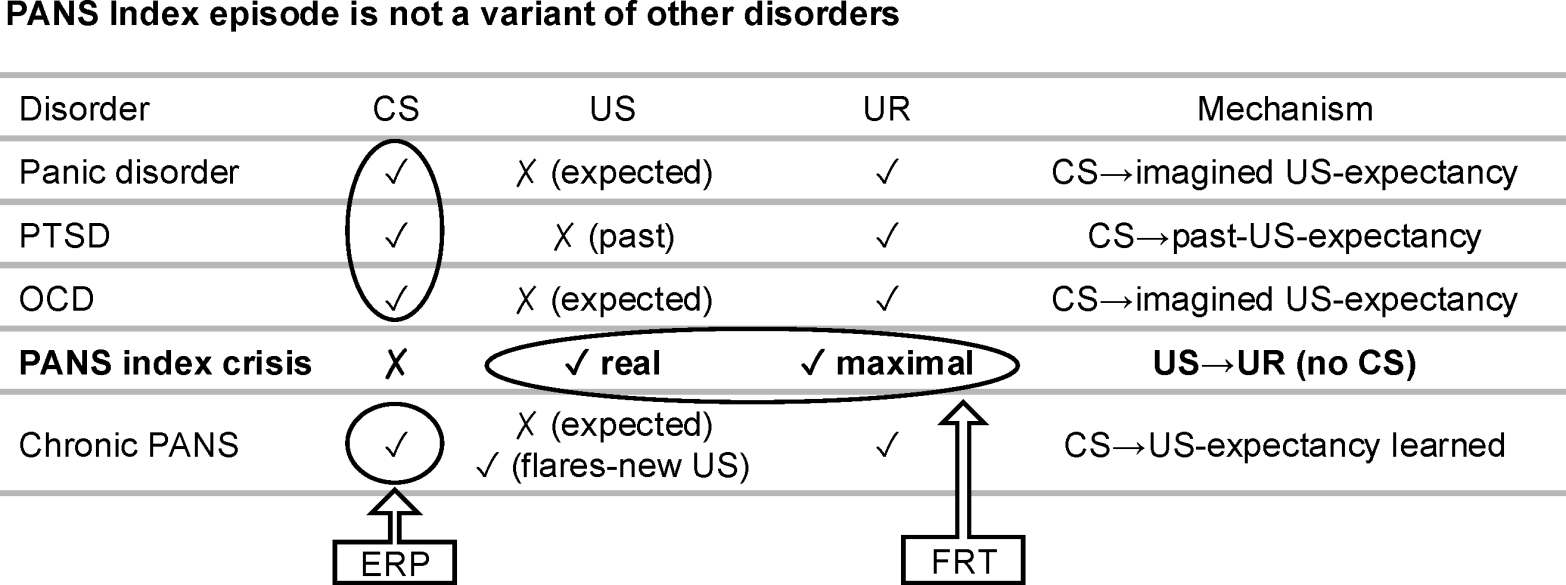
The PANS index episode is not a variant of other anxiety disorders: critical distinctions in learning architecture. Standard anxiety and trauma-related disorders share a common architecture: conditioned stimuli (CS) trigger expectancies of unconditioned stimuli (US), which generate unconditioned responses (UR). In panic disorder, OCD, and PTSD, the CS is present, the US is either expected or past, and treatment targets dismantling CS→US-expectancy associations through exposure and response prevention (ERP). The PANS index crisis presents a categorically different architecture: no CS exists, the US (neuroinflammation-driven fear activation) is present and active, generating maximum UR. This is pure US→UR activation without conditioning history. Chronic PANS, developing after the index episode, acquires CS→US associations and becomes amenable to standard ERP. However, the index crisis itself cannot be treated with CS-based exposure therapy because there is no CS to dismantle. Fear Rebalance Therapy (FRT) addresses this unique unconditioned fear state, while ERP remains appropriate for chronic conditioned presentations.

**Figure 5:**
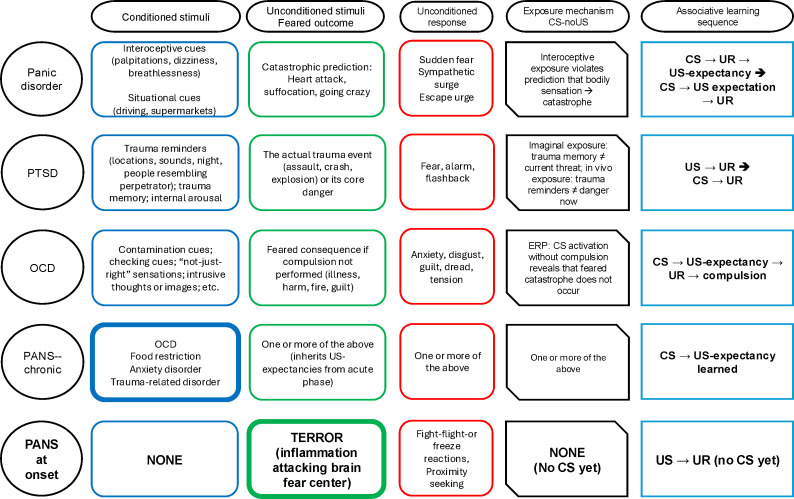
Breakdown of **Figure 4**.

This is unconditioned alarm—the same kind of fear that occurs during a first panic attack or a traumatic event. There is no conditioning history. Just maximum activation of the brain’s threat systems (**Figures 4**, **5**).

### 2.2 Two pathways to fear regulation

Modern exposure therapy operates through inhibitory learning: forming new competing memories that don’t erase the original fear but compete with it during future encounters (10). What optimizes inhibitory learning is not fear reduction during exposure but expectancy violation, deepened extinction, variable practice across contexts, learning frequency, and removal of safety behaviors (11). A co-regulated environment at peak fear maximizes the mismatch between predicted catastrophe and what actually occurs. This is the precise mechanism FRT leverages during the crisis. The theoretical contribution of FRT is that inhibitory learning need not operate at the CS level but can be established at the more primitive US level.

In conventional ERP, inhibitory learning operates on CS-US associations. When a CS is repeatedly presented without the expected US, the individual forms a competing memory (CS → no US). This requires a CS.

**Figure 6:**
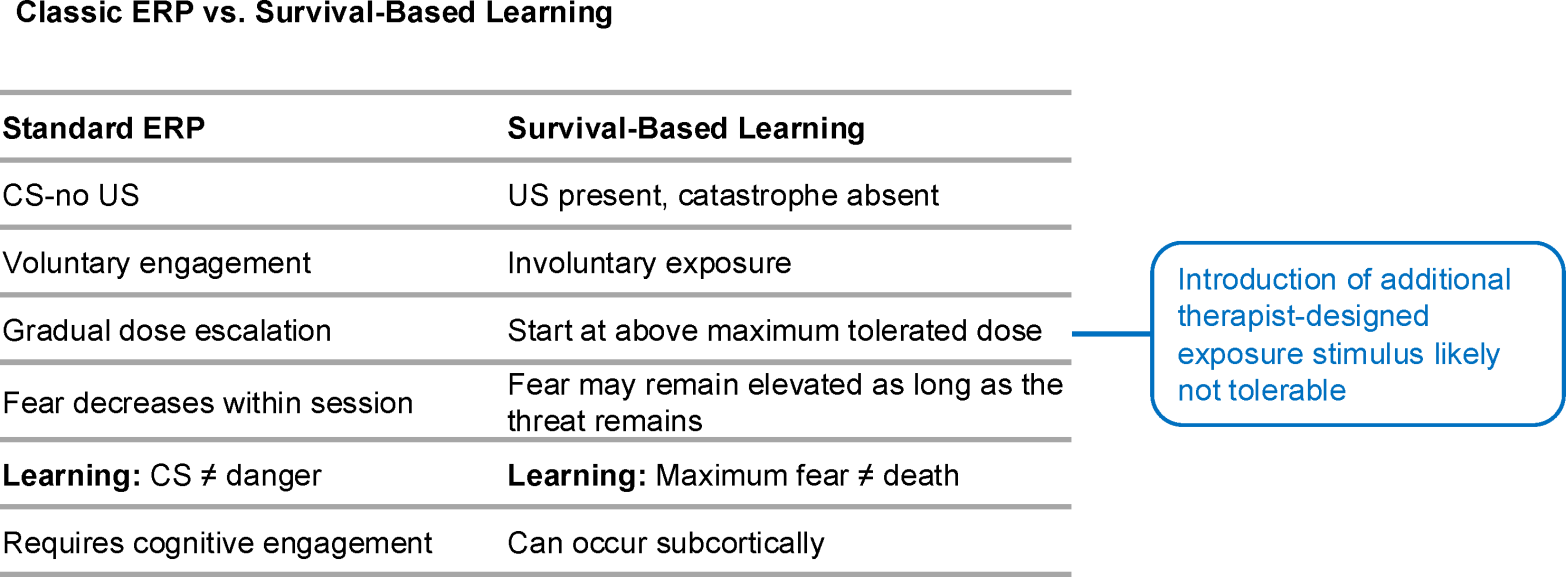
Classic ERP vs. survival-based learning: distinct mechanisms and methods. Exposure and response prevention (ERP) and survival-based learning represent distinct therapeutic mechanisms suited to different fear architectures. Standard ERP targets conditioned fear by presenting the CS in the absence of the US, utilizes voluntary engagement with gradual dose escalation, produces within-session fear reduction, teaches that CS ≠ danger, and requires cognitive engagement. Survival-based learning, central to FRT, occurs when the US is present and active but catastrophe does not occur, involves involuntary exposure beginning at above-maximum-tolerated dose, may maintain elevated fear throughout the threat period, teaches that maximum fear ≠ death, and can proceed subcortically without cognitive mediation. These approaches are complementary rather than competing: survival-based learning prevents sensitization during acute unconditioned fear, while ERP dismantles conditioned associations that may form subsequently.

But inhibitory learning can also operate at a more primitive level: expectancy violation in the absence of conditioning. When threat occurs but predicted catastrophe does not, the individual forms a competing memory: intolerable threat → I survived. This is the learning that occurs during unconditioned fear experiences—first panic attack, index traumatic events, and the PANS index crisis (**Figure 6**).

### 2.3 The trauma parallel

Not all trauma survivors develop PTSD. During a traumatic event, individuals experience maximum unconditioned fear and deploy active defensive responses—fight, flight, freeze, proximity-seeking, hypervigilance. In some cases (combat, for instance), pre-existing CS associations may also be present through training. Yet even with maximum threat activation and sustained defensive behavior, some individuals process the experience in ways that prevent chronic fear sensitization. Co-regulation by stable others, social support, predictable environments, and constitutional factors all contribute to resilient trajectories (12, 13).

PANS follows this pattern. The child experiences sustained internal threat and actively deploys survival strategies—refusing sleep to prevent annihilation, demanding proximity to ensure safety, hypervigilance, heightened sensitivity to sensory stimuli, ritualistic behaviors to control the environment. These are not conditioned avoidance behaviors. They are reflexive defensive reactions to a perceived existential threat (9). Acute stress signaling rapidly impairs prefrontal cortex function, transferring control to the amygdala and making reflective, cognitive engagement temporarily inaccessible (14). Raber and colleagues described the mechanisms of fear learning and memory, showing how the neurobiological state during the crisis determines whether a memory becomes a maladaptive fear association (15).

During the index crisis, the child experiences internal threat with acuity and dramatic severity, and predicts catastrophe. The world will end. Sleep will cause disappearance. Separation will result in permanent loss. Yet repeatedly, these predictions fail. Each terrorizing nocturnal fear ends with morning intact. Despite unbearable fear, the external world remains stable. This constitutes powerful expectancy violation: maximum fear ≠ catastrophe.

The child has an opportunity to encode a survival-based inhibitory trace—not through graduated exposure to a CS (there is none), but through the fundamental experience of facing biological threat and surviving it. This learning occurs in the most primitive terms available to the fear system: I was in real danger. And I am still here.

### 2.4 The two-layer learning in crisis

But survival is not the only thing learned during crisis. The fear system also records incidental cues present during episodes of extreme fear. They may encode protective signals: a parent’s calm presence, stable voice, predictable proximity, environments that remain stable despite internal chaos, experiences where survival is repeatedly confirmed. They may encode sensitizing cues: the child’s own emotions, behaviors, sensory experiences, a parent’s panic or emotional overwhelm, the chaos of emergency rooms, clinicians who invalidate or dismiss their terror. These cues are not intentionally taught, but they get encoded nonetheless.

**Figure 7:**
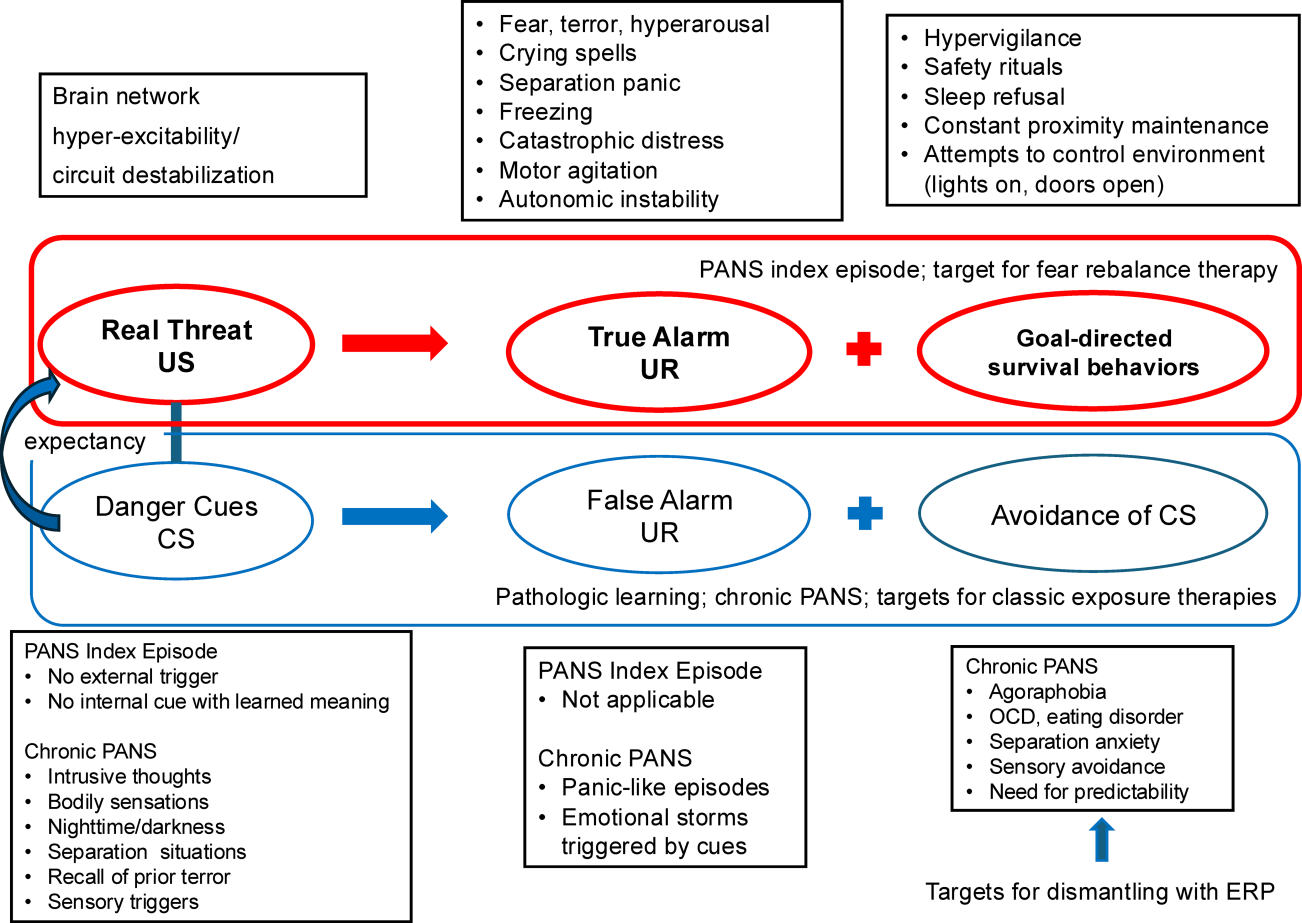
Learning architecture of PANS: index episode vs. chronic presentation. This flowchart distinguishes the learning mechanisms operating during the PANS index episode versus chronic PANS. During the index episode, neuroimmune activation generates unconditioned fear responses (UR) without external triggers or learned cues—a pure US→UR pathway with no CS present. The child experiences true alarms (fear, terror, hyperarousal, crying spells, separation panic, freezing, catastrophic distress, motor agitation, autonomic instability, hypervigilance) and deploys goal-directed survival behaviors (safety rituals, sleep refusal, constant proximity maintenance, attempts to control environment). These are not conditioned avoidance behaviors but adaptive responses to perceived existential threat. This index episode is the target for Fear Rebalance Therapy (FRT). In chronic PANS, CS (intrusive thoughts, bodily sensations, nighttime/darkness, separation situations, recall of prior terror, sensory triggers) → US expectancy has formed, with false alarm. These produce panic-like episodes and emotional storms triggered by learned cues, leading to pathological avoidance patterns (agoraphobia, OCD, eating disorders, separation anxiety, sensory avoidance, rigidity). These conditioned associations are targets for standard ERP. The critical insight: preventing CS formation during the index episode through FRT may prevent the transition from acute unconditioned fear to chronic conditioned fear disorder. Child-friendly version is presented as **Figure 8**.

**Figure 8:**
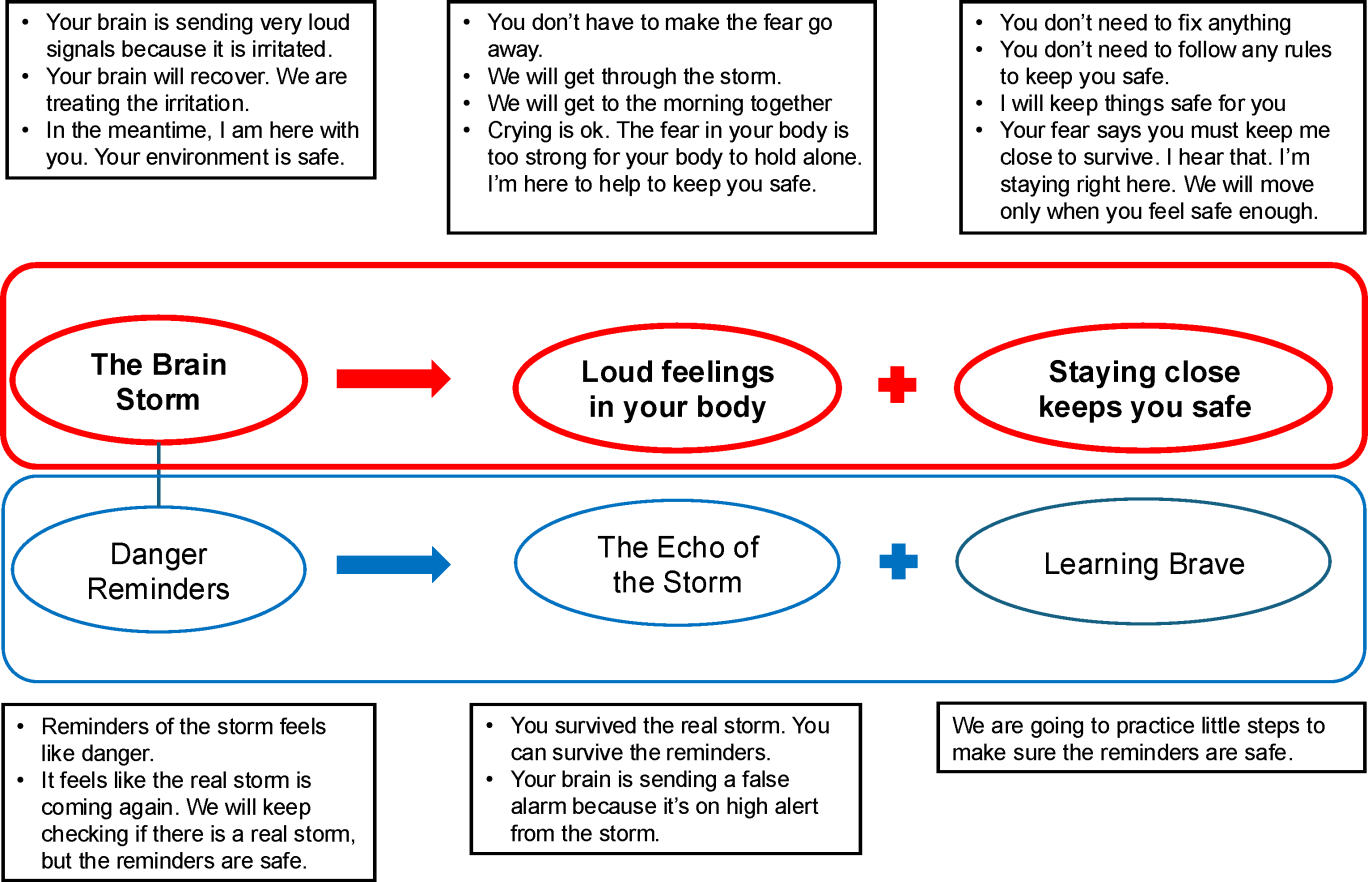
Child-friendly version of **Figure 7**.

**Figure 9:**
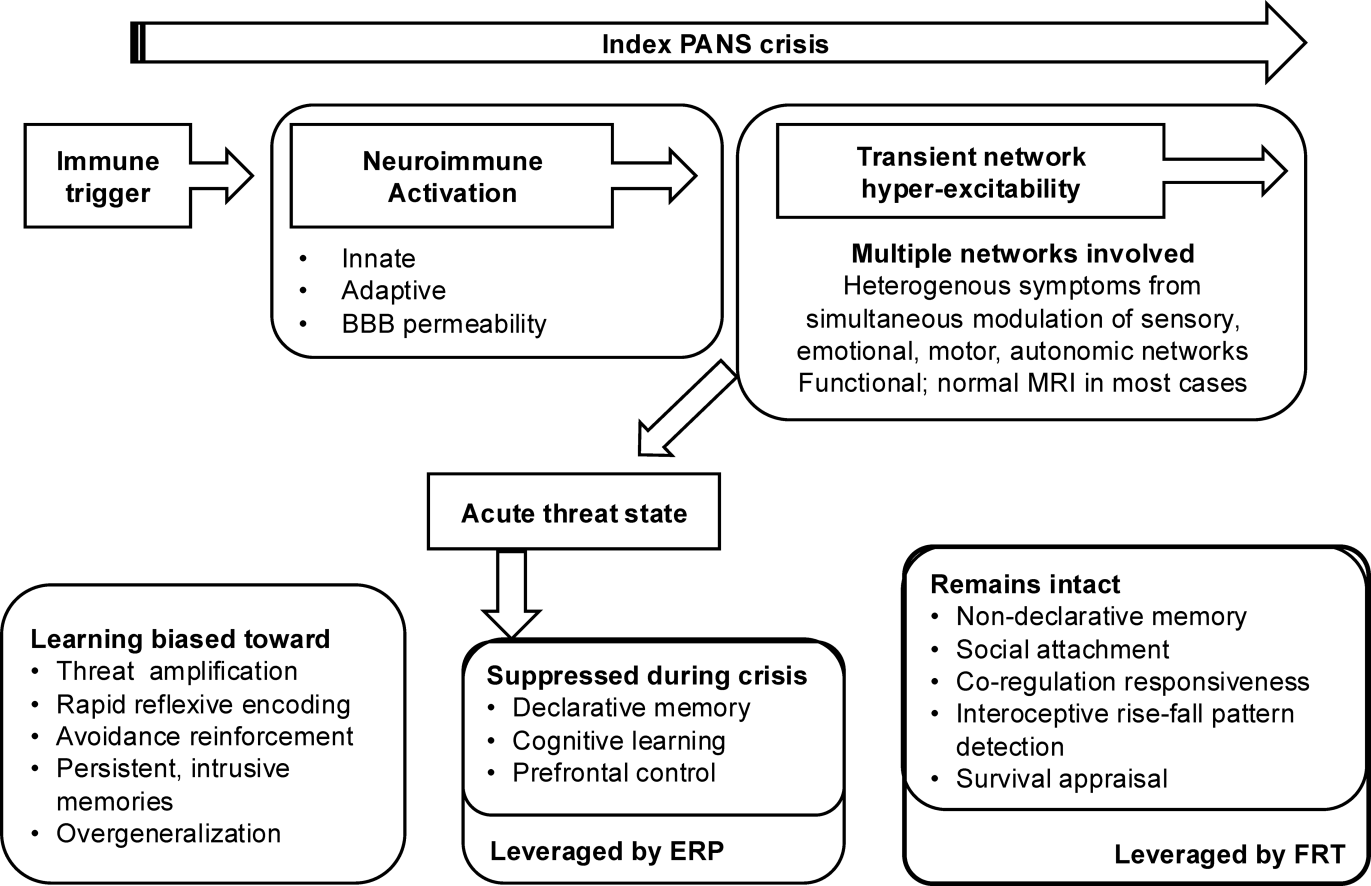
Preserved vs. impaired learning systems during the PANS index crisis. The PANS index crisis results from immune trigger-induced neuroimmune activation causing transient network hyper-excitability. This produces heterogeneous symptoms through simultaneous involvement of multiple brain networks. Networks involved in fear appraisal and threat response are activated (directly or overflow from adjacent networks). Suppressed during crisis: declarative memory, cognitive learning, and prefrontal control—systems typically leveraged by standard ERP. Intact during crisis: non-declarative memory, social attachment, co-regulation responsiveness, interoceptive rise-fall pattern detection, and survival appraisal—systems leveraged by FRT. The acute threat state biases learning toward threat amplification, rapid reflexive encoding, avoidance reinforcement, persistent intrusive memories, and overgeneralization. Understanding this dissociation between impaired and preserved systems explains why cognitive-behavioral approaches requiring declarative processing may fail during acute crisis, while co-regulation-based survival learning remains accessible. FRT is designed to utilize intact systems while avoiding reliance on temporarily compromised cognitive capacities.

The inhibitory trace formed during crisis therefore has two layers: 1. Survival-based learning (maximum fear → I survived), 2. Incidental environmental associations (some stabilizing, others sensitizing). Without intervention, these incidental associations can consolidate into conditioned fear structures. Once CS-US associations have consolidated, ERP becomes appropriate and necessary (**Figures 6**–**9**).

### 2.5 Goal of treatment in the index episode

The goal is not to replace ERP. ERP remains the treatment of choice once conditioned fear structures develop. The goal is to recognize and intervene in the learning environment before those structures form—to help children encode survival and safety during the crisis rather than developing the conditioned fears that later require ERP.

## 3 Fear rebalance therapy

### 3.1 A three-phase framework

**Figure 10:**
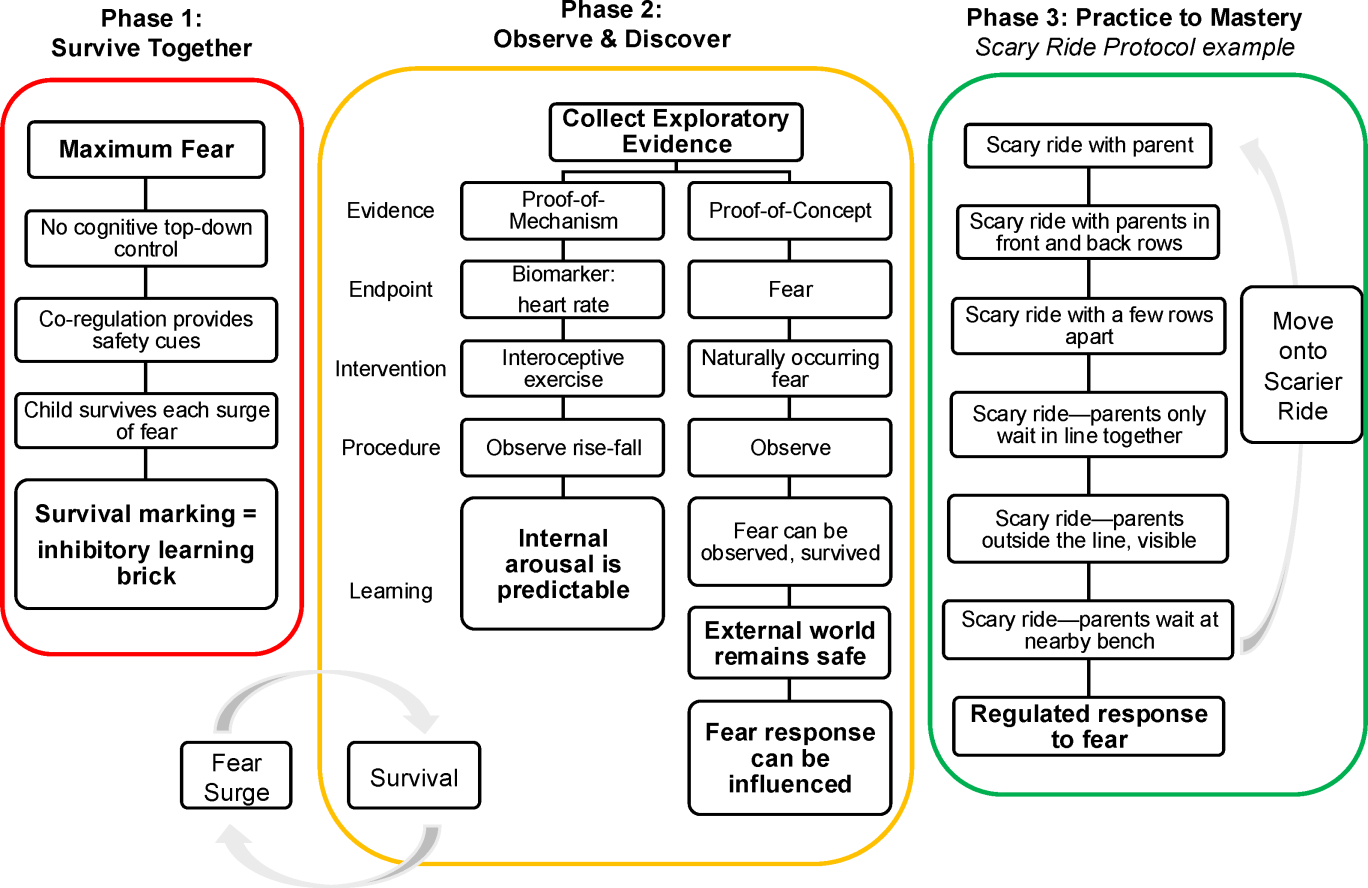
Fear rebalance therapy: a three-phase developmental framework. Fear Rebalance Therapy unfolds across three phases corresponding to the child’s evolving regulatory capacity. Phase 1 (Survive Together) addresses the acute crisis when the child experiences maximum fear without cognitive access. The intervention provides co-regulation as safety cues, enables survival marking of each fear surge as inhibitory learning, and uses the involuntary maximum-dose exposure to establish primitive inhibitory traces: maximum fear does not equal death. Evidence of success is simply surviving together. Phase 2 (Observe and Discover) begins as islands of calm emerge and curiosity returns. The child collects exploratory evidence through biomarker observation (interoceptive exercises demonstrating that heart rate rises and falls predictably) and observes natural fear (fear can be observed, survived, and response can be chosen). This phase provides proof-of-mechanism (internal arousal is controllable) and proof-of-concept (fear response can be influenced). Phase 3 (Practice to Mastery) emerges when fear becomes workable and the child initiates autonomous experimentation. The illustrated “Scary Ride Protocol” shows systematic graduated exposure designed by a child as an example, progressing from maximal parental proximity to independent engagement, then advancing to increasingly challenging fear stimuli. Success produces regulated responses across fear contexts. This phase consolidates flexible inhibitory learning and confirms that the fear system has rebalanced. The framework emphasizes developmental sensitivity: intervention adapts to what the child can access at each phase rather than imposing standardized techniques.

Fear Rebalance Therapy (FRT) operationalizes this goal through a developmentally informed framework that adapts intervention to the child’s changing regulatory capacities across the neuroimmune crisis. The framework organizes the therapeutic stance—what to do and why—across three phases distinguished by different child states, learning mechanisms, and therapeutic tasks (**Figure 10**). The goal of FRT is to prevent maladaptive associative learning during the index PANS crisis and support adaptive inhibitory learning as fear circuitry stabilizes.

FRT is explicitly a psychological intervention targeting fear-learning processes it is not proposed as a treatment for, or alternative to, medical management.

### 3.2 Core therapeutic principle: fear as information, not threat

The foundation of FRT is a consistent, reality-based message delivered across all three phases: Fear is information—it alerts us to potential danger—but the feeling of fear is not a danger in itself.

This framework includes several integrated concepts:

1. Brain’s Alarm System: Fear checks for danger and prepare the body for threat. It is a normal and useful signal.
2. Decoupling Sensation from Harm: The feeling of fear itself cannot hurt you. We don’t need to be afraid of fear. We need to use it to assess our situation.
3. Neuroimmune true alarms to internal threat: During the PANS crisis, neuroimmune activation creates a real internal threat. The fear circuitry responds appropriately to this legitimate danger with enormous signals. While the internal danger is real, the child learns that the external world remains safe and they can survive the internal experience.
4. Reality acknowledgment: Bad things can happen. Death, disasters, and danger are real. We do not dismiss existential truths or gaslight the child’s logical reasoning.
5. Probabilistic appraisal: Existential concerns are addressed by validating possibilities while introducing probability. For low-probability events like natural disasters, the therapist validates that it could happen—like winning a lottery—but emphasizes that the statistical likelihood is extremely low.

Response agency: The child can observe fear, evaluate the situation, and choose how to respond. Fear provides information; the child decides the action. Fear is something to observe and evaluate, not something to fear or obey.

### 3.3 Phase 1: survive the fear together (learn safety of the external world)

#### 3.3.1 Clinical context and child state

Phase 1 corresponds to the height of the crisis, when neuroimmune processes abruptly dysregulates threat appraisal, leading to catastrophic unconditioned fear. Fear often worsens in the evening and peaks at night. Cognitive access is limited: the child cannot sustain rational dialogue, or employ coping strategies. Behavior may include frantic, purposeful-looking attempts to control the environment or prevent perceived existential catastrophe—likely defensive reactions to a true internal alarm.

Fear storms erupt without external triggers, can last hours, and do not habituate because the biological threat is ongoing. The child is undergoing involuntary “above-maximum-tolerated-dose” exposure that cannot be titrated, modulated, or paired with corrective information.

#### 3.3.2 Therapeutic task

The therapeutic task is not to eliminate fear or teach skills. The child’s fear is biologically appropriate given the internal threat state. The task is to ensure that these involuntary fear storms generate inhibitory learning rather than sensitization. The aim is to maintain external safety, prevent overwhelming isolation, and begin laying primitive expectancy-violation traces: maximum fear does not equal catastrophe.

#### 3.3.3 Core interventions

##### 3.3.3.1 Validation of biological reality

Explicitly acknowledge that the fear is enormous and feels catastrophic, and clarify that it originates from real internal danger: brainwide acute state shift, including the fear center, and the brain’s threat response is turned on. The fear response is appropriate to this legitimate internal threat. This can be communicated in age-appropriate language:

Your brain’s alarm system is turned up very high right now. It is working extra hard, and that is why everything feels so scary and overwhelming. We are giving you medications to treat what’s causing it, so it is temporary. The fear you’re feeling is your brain doing it’s job, trying to keep you safe. Fear is information that tells us to check for danger, but we don’t need to be scared of the fear itself. Even though it feels unbearable, the fear feeling itself cannot hurt you. This time, what’s sounding the alarm is inside, not outside. So, you don’t need to do anything to keep yourself safe.

This message can quickly reduce compulsive behaviors once the child accepts that the danger is internal (neuroinflammation) not external, so defensive behaviors targeting the external world cannot address the actual threat. The world remains safe regardless of her defensive actions. Rituals may start to dissolve as this understanding takes hold.

##### 3.3.3.2 Predictive structure

Provide simple, repeated reminders about fear’s temporal course and reinforce the core therapeutic message. Even when the child cannot integrate this cognitively, the repeated message becomes part of the co-regulated environment.

##### 3.3.3.3 Co-regulated presence

The clinician or caregiver maintains calm, steady presence during fear storms. This is neither distraction nor rescue but social buffering: an external regulator when the child’s internal regulation is offline (16–21). The empirical basis for this is substantial. Coan and colleagues (15) demonstrated in a landmark study that holding a partner’s hand measurably reduced neural threat responses, establishing that social contact is not merely emotionally comforting but biologically regulatory. Porges (18) explains that activation of the social engagement system inhibits the fight-flight response, providing a neurophysiological pathway through which calm caregiver presence directly modulates the child’s threat state. Gee and colleagues (20) showed that maternal presence physically suppresses amygdala and cortisol reactivity in children, while Hostinar and colleagues (21) confirmed that this buffering effect is specific to the caregiver relationship and especially pronounced before adolescence—the precise developmental window in which PANS most commonly presents. These findings collectively support the claim that co-regulation during the crisis is an intervention that shapes the learning trajectory.

##### 3.3.3.4 Survival marking

Explicitly mark each survival episode. The morning after a nocturnal terror episode:

*You made it through. Your brain predicted something terrible; morning says survival. This information is now in your system.*

The goal is to create explicit attention to the expectancy violation: maximum fear occurred, catastrophe did not.

##### 3.3.3.5 No deliberate exposure tasks

The child is already undergoing maximum involuntary exposure. Adding structured exposure tasks is unnecessary and potentially counter-therapeutic. The therapeutic focus is containment and survival processing, not behavioral activation.

#### 3.3.4 FRT distinguishes two categories of behaviors

1. Reflexive comfort-seeking (proximity, nightlights, parent in room): These are adaptive defensive responses to perceived existential threat, not learned avoidance. During Phase 1, these should be accepted as the child’s system is already at maximum capacity. Attempting to enforce sleep independence or sleep hygiene during neuroinflammatory crisis is not only ineffective but may amplify distress and prevent the formation of protective inhibitory traces.
2. Perceived survival behaviors (rituals, compulsions, checking): These look purposeful and may be accompanied by the belief that they prevent catastrophe. FRT gently reframes these: “You don’t need rules or rituals to keep you safe. The danger is inside—inflammation in your brain—not outside. I will keep the external environment safe for you.” This plants the conceptual seed that external safety is stable even when internal threat exists, while tactically accepting that maximum arousal may require proximity support.

#### 3.3.5 Learning mechanism

Phase 1 establishes the foundational inhibitory trace: maximum fear does not predict catastrophe. The child cannot yet observe or modulate fear, but through repeated survival marking, co-regulated presence, and the consistent message that “fear is information, not threat,” the primitive expectancy violation begins to register: I felt certain I would die, yet I survived. My brain was under real attack from inflammation, the fear was enormous and appropriate to that internal danger, but the fear itself did not hurt me. The external world remained safe. This trace becomes the substrate for later elaboration in Phases 2 and 3.

### 3.4 Phase 2: observe and discover (proof of mechanism and concept)

#### 3.4.1 Clinical context and child state

Phase 2 begins when neuroinflammation diminishes enough for the child to experience predictable intervals of calm between fear episodes. Markers of readiness include return of curiosity, the ability to observe internal states, asking “why” questions, and seeking explanations. The child is no longer in pure survival mode; islands of cognitive access reappear.

#### 3.4.2 Therapeutic task

The task shifts from containment to collaborative exploration. The child now has partial capacity to observe fear or arousal and begin forming an internal model of its properties and her relationship to these states.

#### 3.4.3 Two complementary learning tracks

##### 3.4.3.1 Artificial arousal experiments (proof of mechanism)

During calm periods, invite the child to engage in simple physiological activities that generate arousal: jumping jacks, running in place, tumbling. The child controls the activity and monitors heart rate, notices breathlessness, then observes these sensations returning to baseline.

These are not classic interoceptive exposures (9). Heart rate elevation holds no conditioned meaning, does not generate fear and functions purely as a biomarker. The child is gathering mechanistic proof: internal arousal rises and falls predictably. This provides a concrete, controllable experience of internal state reversibility.

##### 3.4.3.2 Natural fear observation (proof of concept)

When spontaneous fear episodes occur (now less intense and shorter than in Phase 1), guide the child to observe the fear state without requiring it to resolve. This is not exposure but curiosity-driven data collection: “Notice when the fear starts. Notice how it feels. Let’s watch together and see what happens.”

Reinforce the core therapeutic message. When the child raises existential or probabilistic challenges, validate the logic, acknowledge reality and return to agency:

Yes, bad things can happen. We could both be in danger someday from external disaster. But right now, the probability of that is very low. The danger that’s real right now is inside—the inflammation in your brain. When real external danger comes, we deal with it. But the fear doesn’t help us deal with danger. It just tells us danger might be there.

The child gains evidence that fear can be observed, that safety does not depend on the fear subsiding, and that response to fear can be regulated—it is the child’s decision. This establishes conceptual proof that fear is observable information, that external safety persists independent of internal state, and that agency over response is possible even when arousal remains elevated.

#### 3.4.4 Learning mechanism

Phase 2 transitions the child from a passive victim of uncontrollable fear to an active observer who can evaluate fear as information and choose responses.

#### 3.4.5 Narrative integration (developmentally variable)

For some children—especially those with more sophisticated metacognitive capacity—developing a coherent narrative may facilitate progress. By constructing a conceptual framework involving the brain, immune system, fear circuitry, and neuroinflammation, the child can organize and process an overwhelming experience. The child may ask numerous questions during this process, reflecting cognitive engagement once survival circuitry is no longer overwhelmed. Narrative formation, coherent understanding, and meaning-making contribute to processing the index crisis and reorganizing fear structures (22).

For other children, co-regulation and observation alone may be sufficient. Narrative formation is offered when helpful but not required.

### 3.5 Phase 3: practice to mastery (confirmatory evidence and flexible learning)

#### 3.5.1 Clinical context and child state

Phase 3 emerges when fear becomes “workable”: still uncomfortable, no longer catastrophic, and manageable. The critical transition marker is child-initiated experimentation: the child spontaneously proposes fear-evoking situations (‘What if I try sleeping with the light off?’ ‘What if I try a scary roller coaster?’) rather than simply agreeing to therapist suggestions. This signals restored agency and confidence.

By now the child has accumulated enough exploratory evidence to risk deliberate fear-eliciting tasks. The therapist can gently suggest the framework for this phase and invite the child to participate. The child determines the pace and dose based on readiness.

Fear in Phase 3 is no longer spontaneous. It is experimentally induced in controlled contexts with clear beginnings and endings—much like interoceptive experiments but involving higher-arousal emotional stimuli.

#### 3.5.2 Therapeutic task and intervention

The therapeutic task is to support flexible generalization of inhibitory learning across contexts. Let the child design the exposure experiments while providing a framework: 1) varying intensity, contexts, and levels of parental proximity, 2) predictable boundaries (clear start, end), 3) emphasizing discovery rather than performance.

What appears behaviorally similar to conventional *in vivo* exposure is mechanistically different: the child is not dismantling CS-US associations (though some of these may have formed). Rather, the child is confirming and consolidating the survival-based inhibitory learning acquired in Phase 1, further characterized in Phase 2, and now flexibly applied across domains.

The clinician’s role becomes that of an interested observer, asking, “What did you discover?” The child’s autonomous exploration indicates that inhibitory learning has been successfully internalized and can be self-directed. Children can design their own experiments when given a clear framework. Recent evidence suggests disproportionately higher giftedness in PANS children (23), and PANS clinicians have noted that children come up with creative ways for self-healing.

#### 3.5.3 Learning mechanism

Phase 3 is the confirmatory phase. The child consolidates flexible inhibitory learning through repeated experiments that confirm the child can observe, evaluate, and choose response to fear across varying contexts.

### 3.6 Summary of the three phases

FRT is administered during the index PANS crisis itself—a period often perceived as a therapeutic deadzone until the child becomes “workable” for conventional CBT or ERP. FRT directly addresses pre-reflective fear that is not cognitively mediated. Rather than waiting for the crisis to resolve, FRT provides therapeutic accompaniment during fear storms, explicitly marking survival to establish inhibitory learning in those crisis moments when the child is undergoing involuntary, above-maximum-tolerated-dose exposure.

The framework is organized around the core principle that fear is adaptive information, not a threat in itself. Across three phases aligned with the child’s neuroimmune state and regulatory capacity, FRT supports the development of inhibitory learning: fear does not predict catastrophe, fear can be observed, and the child can choose her response. This approach prevents maladaptive associative learning during the crisis while building the cognitive and emotional infrastructure for long-term fear regulation.

By intervening during—rather than after—the index crisis, FRT transforms the involuntary exposure of PANS into a therapeutic opportunity, leveraging survival experiences to rebalance fear circuitry as it stabilizes.

## 4 Illustrative case

The following case illustrates how the three phases of FRT unfolded during one child’s index PANS crisis and early recovery. Importantly, this framework emerged through therapeutic dialogue with the child herself—her sophisticated reasoning and challenges to oversimplified reassurance shaped the FRT framework.

### 4.1 Phase 1: survive the fear

A previously healthy prepubertal child developed abrupt symptoms at night. The onset was characterized by sudden, frantic, purposeful-appearing behaviors reflecting reflexive attempts to preserve her environment in response to an overwhelming internal alarm. During a later calm session, she articulated the experience as existential threat—an internal sense that catastrophe might happen if she did not act. The compulsive behaviors were disturbing to observe, but for the children, the primary distress was the fear itself.

Daytime remained relatively calm. Symptoms intensified with evening rituals, followed by escalation into maximal nocturnal fear. Anti-inflammatory therapy was initiated within 1.5 days of first symptom onset. Over the first several days, the child endured repeated nights of overwhelming fear, eventually falling asleep from exhaustion. Each morning brought intact survival, marking the primitive expectancy-violation pattern: maximum fear occurred, catastrophe did not.

On day three, the therapeutic message was introduced: “You don’t need to do anything to keep yourself safe.” This message rapidly dissolved the compulsive behaviors within days.

### 4.2 Phase 2: observe and discover

Fear persisted at night, but her cognitive availability during the day was intact. She asked questions, sought to understand what was happening, and pieced together preliminary meaning. She retrieved a sprint analogy given earlier—post-running arousal does not cause fear because it is temporary, controllable, and we know why it happens—and used it as a conceptual anchor. This growing curiosity marked her transition into Phase 2 by the beginning of Week 2.

During this phase, she spontaneously initiated jump-rope experiments, first with her parents and later independently, observing her heart rate rising, remaining elevated, and returning to baseline. These provided proof of mechanism: internal states follow predictable patterns.

In parallel, she began observing natural fear episodes. These nocturnal surges, now shorter and more tolerable, allowed her to notice fear’s temporal pattern: it rose, peaked, and sometimes remained elevated for hours until sleep brought calm. The critical learning was not that fear reliably subsided, but that she could observe fear, remain safe regardless of whether it fell, and choose her response.

As her cognitive capacity returned, she began to challenge the therapeutic reassurance. During a subsequent flare, she stated: “You and I could both die any minute. There might be an earthquake, a sinkhole.” The therapeutic response validated her logic—”You’re right, that could happen”—while introducing probability: “But the chance is super low, like winning the lottery.” This exchange reinforced the core therapeutic message: fear is information about possible danger, but we don’t need to be scared of the fear itself.

For this child, meaning-making was important. She asked detailed questions about the brain, immune activation, and fear circuitry. Understanding “why” introduced predictability and reduced mystification. By the end of Phase 2, her response to fear was more regulated and no longer dominated by panic.

### 4.3 Phase 3: practice to mastery

Over the next few months, she experienced multiple brief infection-related flares. Each started with brief compulsive behavior, which she quickly recognized and resisted, followed by a longer (often throughout the night) but increasingly regulated period of fear. These flares functioned as natural laboratories. Sometimes viral infections did not cause flares. When sequential polymicrobial infections occurred, a brief flare was triggered. She tolerated the unpredictability and was able to describe her experience calmly and matter-of-factly.

After her third flare, she entered Phase 3. She began requesting daily visits to an amusement park and, without external prompting, engaged in natural incremental exposure to fear through scary rides. She looked scared during the rides but laughed afterwards. She varied intensity by adjusting where her parents sat (ride together, adjacent row, wait in line together but ride alone, wait outside the line, wait on nearby bench), making spontaneous moment-to-moment decisions about what she felt ready to tolerate.

Through these deliberate experiments, she confirmed that the principles learned in Phase 2 held true at higher emotional intensities: fear is information, not threat; fear can be observed and tolerated; and she could choose her response without needing to revert to defensive urgency.

### 4.4 The child as active agent in recovery

Children are not passive recipients of intervention but active agents in their own recovery. When provided with therapeutic accompaniment, children not only survive but actively seek their own healing, and may even turn it into play. FRT provides the scaffolding; the child drives the healing process.

### 4.5 Flares as stress tests

The survival memories established during the index crisis create a foundation for progression. When flares occur—and they are common—the child has a competing trace: I survived this before. This allows observation with curiosity rather than catastrophic interpretation.

Each observed flare deepens inhibitory learning: This peaked and passed last time. It will pass again. With each confirmation, confidence grows. Eventually the child experiments: What if I deliberately think about something scary? Can I watch what happens? This is the transition to Phase 3, where the child begins self-directed fear encounters—not because prescribed, but because they’ve developed sufficient confidence to investigate their internal states voluntarily.

**Figure 11:**
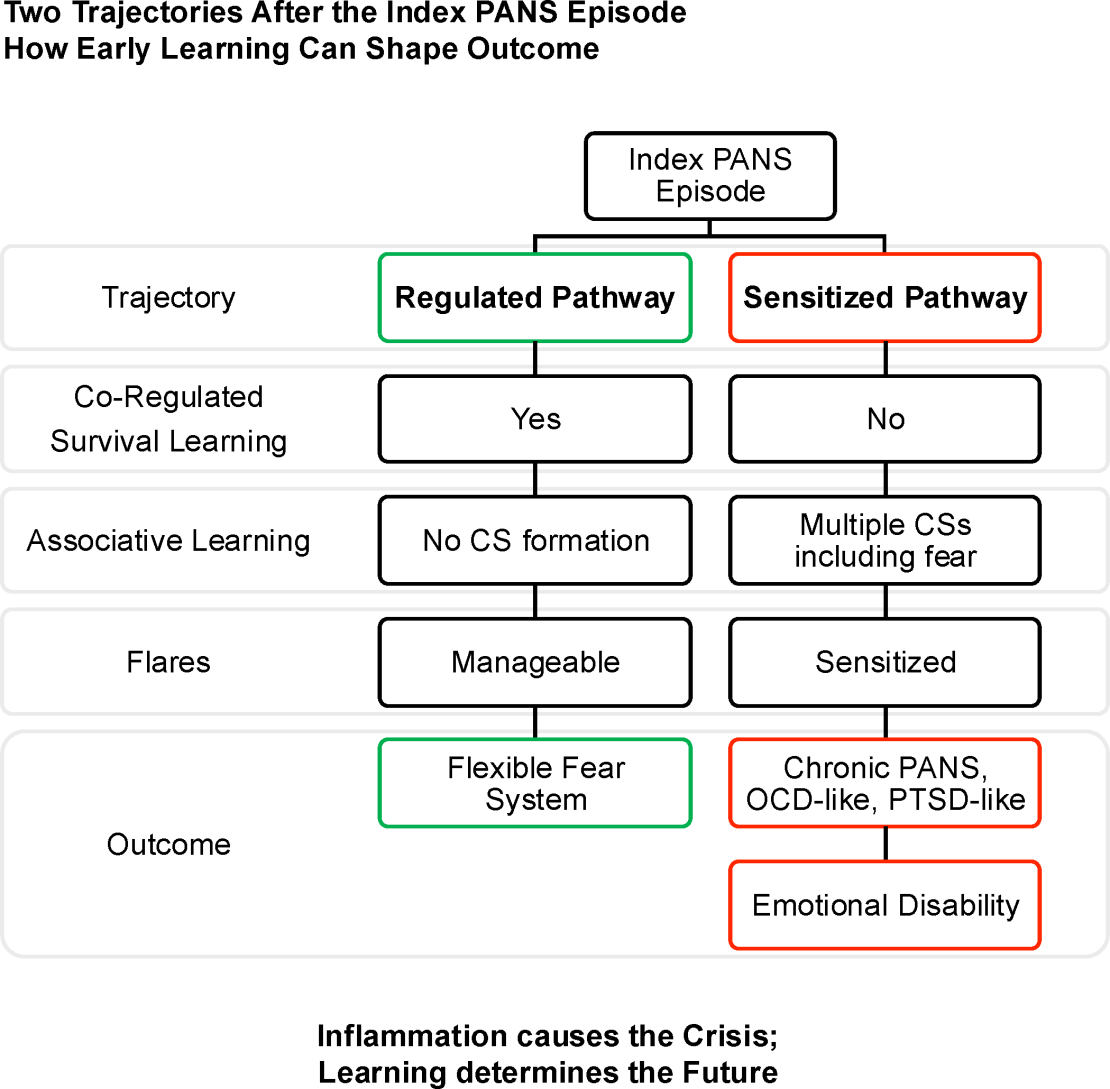
Two trajectories after the index PANS episode: how early learning shapes long-term outcome. The index PANS episode represents a critical branch point determining long-term trajectory. The regulated pathway (left) occurs when co-regulated survival learning is provided during the acute crisis, preventing conditioned stimulus (CS) formation and enabling manageable flares with a flexible fear system. The sensitized trajectory (right) may be taken when the child is left to raw survival, leading to formation of multiple conditioned fear associations (including fear of fear itself), progressive sensitization, and chronic presentations resembling OCD, PTSD, and emotional disability. This framework emphasizes that inflammation causes the initial crisis, but what is learned during the crisis determines the future trajectory. Early psychological intervention is not an adjunct to medical care but a critical determinant of outcome.

What began as neuroimmune catastrophe can, with proper support, become a developmental catalyst. The child learns early that emotions are signals to be evaluated, that responses can be modulated, and that they can control emotions rather than be controlled by them. When supported with developmentally appropriate scaffolding, children can emerge not just recovered but equipped with sophisticated emotional regulation capacities. The same event that could produce chronic sensitization instead becomes an accelerated course in self-regulation (**Figure 11**).

## 5 Managing the triadic relationship

FRT implementation reveals a multi-layered clinical challenge: the child’s neurobiological crisis, parental dysregulation, and systemic pressures.

Parental co-regulation is prerequisite for Phase 1, yet frequently unavailable. Parents oscillate between panic, self-blame, and urgent help-seeking. When the child rapidly attaches to the clinician as the only stable regulatory presence, parents may feel displaced—sometimes generating complex transference. The clinician risks role diffusion and burnout when pulled into surrogate regulator while absorbing parental dysregulation.

Even as the child’s fear system rebalances, parental alarm may remain sensitized. Subsequent minor flares can trigger disproportionate emotional responses that constrain the child’s recovery trajectory (24). Parents cannot provide co-regulated presence or survival marking if they themselves perceive the child’s fear as catastrophic.

FRT therefore cannot be child-only intervention. Explicit acknowledgment of parental dysregulation, early boundary-setting from the initial session (I treat the child; you need separate therapeutic support), and parallel parental support are essential. Clear boundaries protect therapeutic focus and prevent enmeshment in family system dynamics.

Systemic pressures compound the challenge. Multiple clinicians may invalidate the PANS diagnosis, amplifying parental distress. Pragmatically, using comprehensible terminology—acute neuroinflammation, transient inflammation-induced hyperexcitable brain state—may reduce diagnostic conflict while preserving clinical accuracy, allowing diverse providers to anchor around biologically plausible mechanisms without triggering diagnostic skepticism.

Sundowning symptoms in a child may raise questions about the credibility of the reporter. However, once PANS is understood in neuroinflammation framework, it becomes biologically plausible. Sundowning likely results from disruption of normal cortisol–microglial rhythm. Normally, morning cortisol peaks suppress inflammation; at night, microglia rest. Once primed, microglia remain reactive during the nighttime cortisol nadir (25, 26), creating a symptom sundowning window.

## 6 Reorienting familiar skills to an unfamiliar moment

Clinicians already possess the necessary skills for FRT: co-regulation from attachment work, inhibitory learning principles from anxiety and OCD treatment, survival marking from stress neurobiology, narrative formation from emotional processing, and validation paired with reality acknowledgment. The innovation lies not in novel techniques but in recognizing that the index PANS crisis—currently treated as a therapeutic dead zone—is the critical moment to deploy these familiar approaches. The therapeutic target shifts from post-crisis symptom management to acute crisis accompaniment, and the focus shifts from observable behaviors to the child’s internal fear experience.

Clinicians and families who primarily seek practical guidance may find Sections 1–6 sufficient. Section 7 provides the mechanistic and theoretical underpinning for those who wish to examine the hypotheses that inform this framework.

## 7 Mechanistic subtypes and fight-flight-or-freeze framework

### 7.1 Subtype 1: cytokine-driven (febrile seizure-like)

At a high level, both febrile seizures (FS) and PANS can be understood as acute brain-state destabilizations triggered by immune activation. Neither FS or PANS typically shows structural lesion. Rather, both conditions are functional network dysregulations. The driver for this acute disruption is innate immune activation for febrile seizures (27). PANS is defined syndromically, not mechanistically; it likely has multiple mechanisms that converge on OCD networks. A subtype of PANS likely appears innate immune activation-driven. This subtype is tightly temporally related to immune challenges such as infection, immunization or other causes of inflammation such as toxins. It may precede fever by 1–2 days as in FS, episodes are brief, and can have full recovery. NSAIDS shorten flares. The different manifestation of FS and PANS likely stems from children’s brain development stage. Maturity of the cortex, striatum, thalamus, interneurons, synaptic strength likely determine the phenotypic expression of network dysregulation. Just as FS resolves with brain maturation, PANS of this subtype may be outgrown. Immune-modulating treatments such as pulse steroids are likely over-treatment for this subtype. While cytokines are the best-supported mediators of lowered seizure threshold, other innate immune mechanisms may also contribute to network hyperexcitability in this subtype.

Learning, memory involvement is minimal in FS during early childhood, as the fear circuitry and associative learning systems are insufficiently mature to generate catastrophic fear response or encode lasting conditioned associations. Children with PANS, by contrast, are of age where fear circuitry is sufficiently formed while prefrontal regulatory capacity remains immature—the developmental window of maximum vulnerability to both overwhelming fear and pathological fear consolidation. This distinction may explain why FS does not produce trauma, while the index PANS crisis carries significant risk of maladaptive learning.

### 7.2 Subtype 2: adaptive immune-mediated PANS

Some children present with PANS days or weeks after the immune challenge, suggesting adaptive immune involvement. While the field has concentrated on molecular mimicry and B-cell produced antibodies, especially with PANDAS, there may also be patients with T-cell driven pathology. This may explain why peripheral antibody testing may be negative in some patients and treatments aiming to neutralize circulating antibodies are effective in only a subset. Strategies used in other autoimmune brain disorders to reboot the immune system may need to be explored for evolution of treatment algorithms. The adaptive immune timeframe predicts a more chronic state than Subtype 1. These children may show objective neurologic findings and the clinical course is likely to resemble autoimmune encephalitis spectrum illnesses with incomplete inter-episode recovery.

### 7.3 Subtype 3: non-immune allostatic shift

Swedo’s original proposal of PANS acknowledged that acute-onset symptoms can occur in the absence of apparent immune dysfunction (1). We propose that in some of these cases, an existing allostatic shift—a state of neuro-endocrine-immune dysregulation—lowers the threshold for sudden hyperexcitable state shifts. Within this sensitized landscape, even in the absence of an overt immune trigger, the system remains vulnerable to sympathetic overload; this surge can produce neurochemical changes (norepinephrine, dopamine, cortisol) that can predictably impact the cortico-striato-thalamocortical (CSTC) loops (28).

### 7.4 Fight-flight-or-freeze state

Chang and colleagues state that trauma/stress response should be a diagnosis of exclusion for PANS. However, they also describe that during an acute episode, children may appear hyperalert, in the fight or flight mode (2). Although PANS may not be caused by trauma or stress, the index crisis causes extreme stress and can itself be traumatic. Ottaviani & Franceschi referred to antigenic stimuli as non-cognitive stressors, the immune system a sensory organ appraising threat to survival. Antigens and cytokines provoke strong stress responses including cortisol and neuronal excitability (29). These reactions were developed to ensure survival.

The index crisis reflects rapid shifts in brain states that lead to extreme behavioral changes with dramatic acuity. This produces a threat-dominant physiological state. A defense-cascade model is consistent with the multi-system simultaneity observed in the index PANS episode. When the child’s brain detects threat, the brain coordinates autonomic, motoric, attention, attachment, affective and endocrine-immune reactions in a survival configuration that looks like multiple psychiatric and somatic symptoms.

For subtype 1, innate immune activation-induced hyperexcitable state of developmentally vulnerable networks are combined with threat response. Innate immune responses can quickly shape brain state and behaviors (sickness behavior, sleep disruption, cognition/attention changes, autonomic shifts) (30). For subtype 2, adaptive immune mechanisms may cross a certain threshold to elicit the threat-defense cascade. In a review of the threat response, Baldwin emphasized that the reactions are inherently psychobiological and that symptom variability follows shifts among autonomically distinct defensive states such as freeze-alert, fight, flight, freeze-fright, collapse (9). A reconceptualization of symptoms in the initial PANS crisis as URs or US-driven defensive reactions is presented in **Table 1**. Nearly all symptoms are UR-like automatic, non-volitional reactions (e.g. motoric overflow), purposeful-appearing defense reactions as survival strategy (e.g. safety rituals), or a mixed UR + defensive reaction (e.g. rituals that are both motor overflow and serve survival strategy). The child has perceived danger and produced defensive outputs that resemble psychiatric syndromes, but function like survival behaviors. When viewed in this context, the approach to symptoms in FRT Phase 1 (e.g. not blocking rituals, but gently introducing that rituals are not necessary to keep the environment safe) could be better received.

**Table 1:** Reconceptualizing PANS symptoms as defensive cascades and key intervention principles for initial PANS index crisis.

Symptom clusters	Index crisis/flares: defense-state interpretation	Intervention (FRT phase 1)
Obsessive–compulsive symptoms	Hypervigilant threat monitoring and motoric discharge (alert, fright); rituals function to preserve safety or prevent catastrophe	Do not block rituals; “you don’t need to do anything to keep the word safe”; avoid ERP; reduce threat cues
Food restriction/ARFID-like symptoms	Early: sickness behavior (nausea, anorexia), interoceptive threat, fear of swallowing/vomiting (freeze-fright)	Prioritize nutrition and medical safety; no exposure-based feeding
Separation anxiety	Primitive attachment-based proximity seeking under; caregiver functions as external regulator	Permit proximity; co-regulation; no separation training
Generalized anxiety/panic	Threat-dominant physiology with narrowed attentional scope; anxiety reflects state, not cognition	Validate fear; minimize demands; avoid cognitive restructuring
Emotional lability (crying spells, meltdown)	• Limbic overload; fear exceeds child’s regulatory capacity • Crying as distress signaling (“I cannot handle this alone”)	**Steady co-regulation**; validation; quiet environment; “fear rises/fear falls”; no behavioral correction
Aggression/rage	Fight response; cortical inhibition compromised	Ensure safety; neutral containment
Motor hyperactivity/tics	• Motor discharge from inflamed sensorimotor loops • Defensive scanning	**Reduce sensory load**; gentle containment; weighted blankets if tolerated; *no* behavioral shaping; maintain safety
Regressive behaviors	• Threat-induced developmental fallback (freeze-fright or collapse) • Seeking simpler, safer modes of interaction	Simplify demands; **supportive scaffolding**; avoid pushing independence; stable structure
Sensory hyperreactivity (textures, light, sound)	• Sensory gating failure • Hypervigilance: orienting to every sound/light as potential danger	Reduce sensory input; predictable environment; avoid sensory exposures; co-regulate through surges
Somatic symptoms	• Urinary urgency, enuresis: autonomic, brainstem, neuroendocrine dysregulation • Autonomic response (headache, abdominal pain, fatigue)	**Reassurance**; maintain routines; avoid shame; medical check but emphasize temporary nature
Sleep-refusal, night terror, fear of dark	• Night terrors may be limbic arousal bursts • Sleep refusal: prevent catastrophic annihilation by staying awake	**Night-support protocol**: stay in room or nearby; reduce darkness-related cues; no sleep training; structured breathing routines; **morning survival → inhibitory learning**
Cognitive dysregulation (brain fog, attention drop, executive failure)	• Network overload • Cognitive resources diverted to survival; executive function offline	**Academic hold pattern**; no demands; simple tasks; continuity routines

In the initial PANS crisis, nearly all symptoms are URs or US-driven defensive reactions, not CS-learned fear responses. Bold text highlights important actions.

This framework is etiology-agnostic, phenomenology-driven, and phase-specific (crisis phase). How we address this phase aligns with how psychiatry already treats other acute states. The crisis phase is a physiologic state shift, not immediate onset of a psychiatric disorder. It does not yet meet the assumptions required for stable psychiatric disorders until maladaptive associative learning consolidates or the threat state is chronically sensitized.

When symptoms persist beyond FFFS, now it is time to address them like how psychiatry treats chronic disorders. Persistent obsessions/compulsions decoupled from flares now need ERP/CBT. Conditioned avoidance of foods/textures/contexts needs exposure-based feeding therapy. Anticipatory anxiety and safety behaviors need CBT. Conditioned insomnia needs sleep hygiene.

Flares are state reactivations and the presentations are shaped by how the crisis phase and previous flares were dealt with.

### 7.5 Individual clinical applications

The subtypes and FFFS framework described above operate at a conceptual level, identifying triggering mechanisms and the common downstream threat-state they converge on. In clinical practice, however, how FFFS manifests and how severe and prolonged it becomes is shaped by two individual-level modulators: the child’s neurodevelopmental stage and their pre-existing biopsychosocial vulnerabilities. Understanding these modulators is essential for tailoring FRT to the individual child and for identifying those at highest risk for pathological fear consolidation.

The three subtypes are not mutually exclusive—multiple combinations are possible, as shown in **Table 2**. Importantly, this model suggests that PANS may be less dependent on the nature of the trigger than on the child’s capacity to resolve the insult. When immune load exceeds neuro-endocrine buffering capacity, whether through a single overwhelming trigger or the convergence of multiple subtypes, the system shifts into a threat-dominant state. The trigger initiates the crisis; the child’s capacity to resolve it determines the course.

**Table 2:** Possible subtype combinations in PANS.

Subtype 1 (innate immune)	Subtype 2 (adaptive immune)	Subtype 3 (allostatic shift)	Clinical implication
Yes	No	No	Brief, self-limiting, full recovery likely
No	Yes	No	Chronic; incomplete inter-episode recovery
No	No	Yes	No immune trigger; medical/psychiatric background likely
Yes	Yes	No	Acute onset evolving to more chronic course
Yes	No	Yes	Acute immune crisis amplified by pre-existing vulnerability
No	Yes	Yes	Chronic autoimmune course with lowered threshold for hyper-excitable state shifts
Yes	Yes	Yes	Chronic neuro-endocrine-immune dysregulation amplifying response to immune challenges

This combinatorial model explains the substantial heterogeneity in PANS presentations and trajectories observed clinically.

#### 7.5.1 Effects of age and sex

PANS is a developmental window disorder, classically presenting in pre-pubertal children, and FRT is conceptualized for this period of life. During childhood, amygdala-prefrontal connectivity is immature—the two structures are positively coupled, meaning they amplify rather than regulate each other. Gee and colleagues (31) demonstrated that this shifts fundamentally during adolescence, when inhibitory coupling gradually develops and top-down prefrontal control over fear becomes available. In pre-pubertal children, this inhibitory architecture does not yet exist. Fear, once activated, is experienced without the cortical braking system that would later allow modulation. This is why the FFFS response in PANS seems to take on such catastrophic, unmodulated intensity in this age group, and why co-regulation by an external adult serves as a necessary substitute for the prefrontal inhibition the child’s brain cannot yet provide. In this line of thought, late adolescent-adult *de novo* onset of PANS becomes less likely without Subtype 3, as the maturing inhibitory architecture provides increasing buffering capacity against fear escalation. The progressive maturation of the blood-brain barrier as a regulator of peripheral-central immune signaling contributes further to this age-dependent buffering (32).

Within childhood, which neurodevelopmental processes are most actively underway at the time of PANS onset may further shape symptom expression. Networks under active construction, not yet fully consolidated, in their dynamism may make them most susceptible to disruption. A child in the language acquisition period may express FFFS disruption through regression to baby talk; one in the fine motor refinement period through dysgraphia; one in the identity formation period through anorexia-like restriction. The dominant neurodevelopmental process does not cause the symptom — the FFFS state does — but the most actively developing networks may be the most susceptible to its expression.

In the FFFS framework, OCD may be understood as a proactive fight response—high-energy, purposeful-appearing attempts to exert agency over an invisible internal threat. Rituals in pre-pubertal children are likely driven by concrete operational and externalized logic, such as a specific action is required to prevent a parent’s death. Adolescents with formal operational logic and identity maturation may turn the same fight imperative inward, targeting one’s own mind, moral standing, or sense of self.

Sex differences in FFFS behavioral output likely exist as well; girls may tend to be driven by internalized scanning and boys more likely to externalize the fight responses. However, effects of sex steroids on the brain are complex, additionally modulated by ovarian cyclicity in girls (33), and require dedicated investigation.

#### 7.5.2 Pre-existing biopsychosocial vulnerabilities

We define Subtype 3 as an acquired state of neuro-immune dysregulation where the threshold for CSTC loop disruption is significantly lowered. This allostatic shift is frequently rooted in the child’s pre-existing vulnerabilities.

##### 7.5.2.1 Biological vulnerabilities

The most clinically recognizable biological vulnerabilities are pre-existing immune disorders or recent infections that disturbed immune homeostasis. Conditions or treatments that impair cortisol inhibition of inflammation represent another vulnerability category, e.g. disruption of the HPA axis leading to functional adrenal insufficiency compromises the neuro-endocrine-immune system (32). Periods of significant endocrine flux, such as growth spurts or pubertal transition, introduce additional hormonal variability at the neuro-endocrine-immune interface. Treatments or conditions affecting neurosteroid levels will alter excitatory or inhibitory neuronal signaling (33). Even *in-utero* events such as maternal infection may act as an early allostatic driver (34).

##### 7.5.2.2 Psychosocial vulnerabilities

The child’s relational and environmental landscape (e.g. criticism, hostility, and emotional over-involvement) may act as a chronic trigger sustaining a background state of neuro-endocrine activation that lowers the threshold for system failure when immune challenge arrives. Hyperarousal states in ADHD or anxiety disorders, sensory processing disorders, and a history of childhood adversities compound this vulnerability. Felitti and Anda demonstrated that accumulated adverse childhood experiences are associated with chronic inflammatory and autoimmune conditions (35), a finding that situates psychosocial adversity not merely as psychological background noise but as a biological modifier of immune regulation.

Biological and psychosocial vulnerabilities converge on the same neuro-endocrine-immune interface, each contributing to the cumulative allostatic load that determines whether an immune challenge tips the system into crisis.

## 8 Measurement, monitoring and path toward an evidence-based practice

Operationalization into a measurable evidence-based protocol is an important next step. The Children’s Yale-Brown Obsessive Compulsive Scale (CY-BOCS) has been used as outcome measures in PANS ERP trials, offering validated, parent-meaningful tool that tracks the most behaviorally visible symptom. Given the rapid change in severity, characteristic of PANS, a timeframe modification to within the past 24 hours, rather than the standard one-week, was clinically more informative. Subjective Units of Distress (SUDs) proved useful for tracking compulsion-related distress during the child’s attempts to resist. Tracking SUDs to de-escalation helped restore early agency. The Patient Global Impression of Severity and Change (PGI-S and PGI-C) may be useful as complementary measures.

### 8.1 Monitoring the child’s experience of fear

While compulsive behaviors are most visible to observers and most amenable to standard measurement, for the child, the central experience is fear. Before discussing specific instruments, it is worth naming what clinical improvement actually looked like in practice. Fear during the index episode did not fluctuate unpredictably. It followed a predictable circadian pattern, creeping upward as night emerged and becoming effectively binary: maximum intensity from onset until sleep, absent upon waking. SUDs could not meaningfully engage with fear in this threshold state; once the child crossed into the nocturnal fear window, everything registered at maximum, and the instrument had nothing left to differentiate.

What changed over time and across subsequent episodes was the shape of the fear curve. Three dimensions of improvement were observed: peak intensity blunted, duration shortened, and the escalation slope shallowed. Taken together, these constitute a change in total fear burden: the area under the curve (AUC) of intensity and duration. This trajectory was visible to the clinician and recognized by the child in retrospect. Total fear burden (fear AUC) may therefore be the most clinically meaningful outcome marker for the acute phase. Future research should develop measurement approaches sensitive to this threshold and trajectory pattern.

### 8.2 Assessing prevention of pathological fear conditioning

As seen with other disorders involving maladaptive learning, fear sensitization will likely lead to increase in daily total fear burden with each subsequent flare. If FRT successfully prevents such maladaptive fear learning and allows acquisition of fear processing skills, the total fear burden during flares will decrease, as well as fear peak.

Prevention of pathological fear consolidation can be additionally operationalized as the absence of new CS–US association formation following the index episode—specifically, the absence of conditioned fear responses to cues incidentally present during the crisis (e.g., darkness, certain places, smell, sound, social situations) that were not feared prior to it. PANS is well-suited to this assessment because the index episode has a relatively clear onset, unlike cumulative developmental trauma, making pre/post comparison feasible.

This construct has a detection problem: it is inherently easier to measure what happened than what did not. Absence of conditioning must be inferred rather than directly observed. Systematic documentation of all contextual cues present during a crisis is not feasible; however, some can be identified when the child verbalizes or behaviorally communicates new fear of specific stimuli. Whether these have acquired fear valence, or whether the child shows avoidance of mapped cues, constitutes a clinically meaningful measure. Parents and children are likely to offer complementary reporting: parents are generally better reporters of observable avoidance behavior, while children are better reporters of interoceptive cues and subjective fear. Incubating conditioned responses not yet expressed in behavior cannot be assessed by either reporter. Rates of ERP-eligible conditioned fear disorder at follow-up (e.g., CS-present OCD, specific phobia, PTSD, various anxiety disorders emerging after the index crisis) represent a further measurable downstream outcome.

Whether FRT has benefit over unsupported PANS episodes would be supported by evidence showing that children demonstrate: (1) lower fear AUC trajectory during subsequent flares, (2) fewer new conditioned fear associations at one, six, and twelve months post-episode, and (3) lower rates of ERP-eligible conditioned fear disorders at follow-up.

The proposed conceptual framework would be falsified if children receiving FRT show rates of chronic conditioned fear disorder equivalent or higher than those who received no such support. It would also be weakened if incidental conditioning during the acute crisis is found to be negligible, but this is unlikely given clinician observations as well as the patient descriptions in the PANS ERP studies discussed above.

The feasibility of conducting well-controlled studies is constrained by the rarity and abruptness of PANS onset and the need to match developmental stage, biological subtype, and psychosocial characteristics across conditions.

## 9 Limitations and future research directions

This is a conceptual model, not an evidence-based protocol. There may be several challenges to the implementation. Most importantly, the clinical reality is that many children will not get recognized as candidates for FRT during the window of maximal inhibitory learning opportunity.

The illustrated case provides proof-of-concept under highly favorable conditions. The child had immediate diagnostic recognition, early anti-inflammatory treatment, extensive specialist access, high cognitive functioning, and an attached extended family—factors that limit generalizability. We cannot disentangle the contributions of anti-inflammatory medication, therapeutic relationship, specific FRT techniques, spontaneous remission, or other supportive factors.

The treating psychiatrist’s professional background (eating disorders unit where ERP is in the treatment milieu, combat PTSD exposure therapy, pharmaceutical development in cognitive impairment and pain central sensitization) may have systematically shaped this framework. Clinicians with different backgrounds might conceptualize acute PANS intervention very differently.

The child who inspired this treatment model had preserved metacognitive capacity during crisis. Whether FRT principles apply to children with severe cognitive impairment, younger developmental stages, or co-occurring neurodevelopmental conditions remains unknown.

The intensive clinician involvement required for Phase 1 raises questions about scalability and sustainability. Over time, parents and grandparents were able to provide co-regulatory presence, and implement survival marking. Whether family can perform these tasks in the immediate crisis without intensive clinician support requires investigation. However, given accounts of numerous parents persevering to seek healing for their children, family-led implementation appears feasible.

Future research should address several questions:

1. Does early co-regulated support during acute PANS predict better long-term trajectory compared to standard supportive care or watchful waiting?
2. Can survival marking be effectively implemented by trained parents without intensive clinician involvement?
3. What are the essential versus optional components of FRT?
4. Can FRT work during flares when true alarms (immune activation) and false alarms (conditioned cues) fire simultaneously?
5. Does FRT reduce rates of chronic psychiatric sequelae (OCD, anxiety disorders, PTSD) compared to standard care?
6. Do the conceptual subtypes hold in clinical practice?
7. How can we best measure clinical change?

## 10 Conclusion

Children experiencing the index PANS crisis do not start at the bottom of an exposure ladder. They begin at the top: catastrophic, unrelenting fear driven by neuroimmune activation—maximum involuntary exposure without a conditioned stimulus to target, without a window for habituation, and without the cognitive architecture that conventional ERP requires.

Current clinical wisdom holds that they are ‘too ill’ for psychological intervention. This perspective, while understandable, represents a missed therapeutic opportunity. The child is already undergoing exposure—above-maximum-tolerated-dose exposure. The question is not whether to introduce exposure, but whether these fear storms generate inhibitory learning or chronic sensitization.

Fear Rebalance Therapy reframes the crisis as the critical intervention window—the moment when foundational learning occurs or fails to occur. By applying established principles from inhibitory learning theory, stress biology, trauma treatment, and developmental psychopathology to unconditioned fear states driven by neuroimmune activation, FRT proposes that survival-based expectancy violation can generate primitive inhibitory traces that outcompete maladaptive associative learning. These traces, formed during the acute crisis through co-regulated presence and survival marking, may protect against the conditioned fear structures that would later require ERP to dismantle.

This remains a conceptual framework requiring validation. FRT has not been tested in controlled studies; the argument presented rests on theoretical reasoning from established learning science, stress biology, and developmental psychopathology, together with clinical observation. However, the clinical logic is compelling: our role is not to wait until the storm passes. Our role is to help the child’s brain learn what the crisis itself reveals: fear can come and fear can go. And the world remains intact—regardless of how they react to it.

If this framework proves valid, it positions psychological intervention as an active component of acute PANS crisis management rather than a post-crisis adjunct. The goal is not symptom elimination during neuroimmune storm—the biological process must run its course. The goal is shaping how the storm is encoded: as trauma that produces chronic fear sensitization, or as survival that teaches emotional regulation.

With proper support, what might have produced a lifetime of psychiatric morbidity may instead produce early mastery of what many adults never learn: emotions are information to be processed and regulated. The capacity to regulate one’s response to fear can be discovered, not taught.
